# Supplementary material for: Genome-wide association identifies novel ROP risk loci in a multiethnic cohort
Source: Commun Biol. 2024 Jan 17;7:107. doi: 10.1038/s42003-023-05743-9 (PMC10794688; doi:10.1038/s42003-023-05743-9)
Supplement: Supplementary file 2 — Supplementary Information [file 42003_2023_5743_MOESM2_ESM.pdf]

Supplementary Data

Gene Name	SNP	Relationship	Reference	Study Design	Race/ Ethnicity	N	Outcome Measure	Analysis Design	Significance	BW and GA adjustment
BDNF	rs7934165	risk	Hartnett et al; 2014	Case control	71.4% self-reported black	Control: 264 Mild ROP: 467 Severe ROP: 126	Severe ROP	Candidate SNP Chip-based	$P = 2.9 \times 10^{-7}$ in meta-analysis	Yes
BDNF	rs2049046	risk	Hartnett et al; 2014	Case control	71.4% self-reported black	Control: 264 Mild ROP: 467 Severe ROP: 126	Severe ROP	Candidate SNP Chip-based	$P=3 \times 10^{-5}$	Yes
THSD4		risk	Kim SJ et al; 2021	Case control	Non-Hispanic Caucasian	58 Severe ROP 42 Mild ROP	Severe ROP	Whole exome sequencing	$P = 5.04 \times 10$-^{4}	Yes
BDNF	rs7929344	protection	Ilguy S et al;2021	Case control	Persian	75 mild-moderate ROP 73 severe ROP	Severe ROP	Candidate SnapShot technique	$p < .001$	No
VEGFA	rs2010963	risk	Ilguy S et al;2021	Case control	Persian	75 mild-moderate ROP 73 severe ROP	Severe ROP	Candidate SnapShot technique	$p < .001$	No
VEGFA	rs3025039	risk	Ilguy S et al;2021	Case control	Persian	75 mild-moderate ROP 73 severe ROP	Severe ROP	Candidate SnapShot technique	$p < .001$	No
EPAS1	rs13419896	risk	Ilguy S et al;2021	Case control	Persian	75 mild-moderate ROP 73 severe ROP	Severe ROP	Candidate SnapShot technique	$p = .022$	No
NOS3	rs2070744	risk	Ilguy S et al;2021	Case control	Persian	75 mild-moderate ROP 73 severe ROP	Severe ROP	Candidate SnapShot technique	$p = .004$	No
VEGF	460 T/C	risk	Luo Y et al; 2019	N/A	N/A	14 studies	Presence or absence of ROP	Meta-analysis	C vs. T, OR = 0.83, 95% CI: 0.74–0.94, P_{OR}=0.004	N/A
TNF -308G/A	308G/A	Risk	Lei XJ et al; 2018	Case control	Chinese	724 advanced ROP	Presence or absence of ROP	Candidate PCR-RFLP method	GA genotype, aOR: 1.956, 95%CI: 1.396-2.465,	Yes reflected in adjusted OR

						878 controls		with sequencing validation	P<0.001; AA genotype, aOR: 2.809, 95%CI: 1.802-4.484, P<0.001	
ACE ID	D allele	protection	Lei XJ et al; 2018	Case control	Chinese	724 advanced ROP 878 controls	Presence or absence of ROP	Candidate PCR-RFLP method with sequencing validation	ID genotype, adjusted OR (aOR): 0.603, 95%CI: 0.427-0.893, P=0.034;<br><br>DD genotype, aOR: 0.468, 95%CI: 0.229-0.626, P=0.002	Yes reflected in adjusted OR

Supplementary Table 1: Prior Evidence of Genetic ROP Risk

Race/Ethnicity	No ROP	Stage 1	Stage 2	Stage 3	Stage 4	Stage 5	Total
**European Ancestry**	370	174	198	179	35	11	967 (44.2%)
**African American**	123	33	44	47	3	1	251 (11.5%)
**Hispanic**	246	135	235	149	49	43	857 (39.2%)
**Other**	46	14	26	20	1	5	112 (5%)
**Total**<br>**%**	785<br>(35.9%)	356<br>(16.3%)	503<br>(23%)	395<br>(18.1%)	88<br>(4%)	60<br>(2.7%)	2187

Supplementary Table 2: Full iROP cohort ethnicity and ROP diagnosis

SNP	chr:position	Ethnicity	Effect allele	AF: Case	AF: Control	OR	p-value	p-value adj. BW & GA	gene
rs17048572	4:137201813	HA	C	0.02	0.02	Na	Na	na	none
		EA	C	0.14	0.04	1.39	4.19E-5	1.99E-8*	
		AA	C	0.04	0.06	0.88	0.40	0.15	
		All	C	0.06	0.04	Na	Na	na	
rs9978278	21:37894721	HA	T	0.06	0.09	0.96	0.88	0.48	*CLDN 14*
		EA	T	0.12	0.24	0.94	5.8E-03	0.08	
		AA	T	0.25	0.03	1.71	4.04E-9*	1.63E-8*	
		All	T	0.09	0.13	0.97	0.16	0.24	
rs74048122	14:43889545	HA	G	0.03	0.06	Na	Na	na	None
		EA	G	0.08	0.07	0.99	0.55	0.86	
		AA	G	0.32	0.04	1.53	2.62E-8*	3.14E-8*	
		All	G	0.07	0.06	1.03	0.25	0.43	
rs1004464	9:131003677	HA	G	0.06	0.13	0.93	0.17	0.14	*DNM1*
		EA	G	0.13	0.18	0.95	0.40	0.20	
		AA	G	0.61	0.19	1.29	1.74E-7	2.62E-8*	
		All	G	0.14	0.17	0.99	0.86	0.82	

Supplementary Table 3: Racial and Ethnic differences in GWAS-identified SNP associations with severe ROP disease

Supplementary Table 4: Top regional *GLI3* SNPs by Ethnicity and Race

SNP											
SNP rs2058019	Chr	pos	Ethnicity/ Race	Effect Allele	AF	AF: Case	AF: Control	OR	p-value (with BW & GA adjustment)	p-value (no BW & GA adjustment)	gene
	7	42226712	EA	T	0.012	0.037	0.006	1.61	4.00E-04	4.40E-03	*GLI3*
			HA	T	0.113	0.218	0.078	1.26	2.95E-06	1.10E-05	
			AA	T	0.014	0	0.015	0.87	0.49	0.37	
rs3779167			All	T	0.06	0.135	0.039	1.27	4.96E-09*	5.25E-08	
	7	42217416	EA	A	0.011	0.037	0.004	1.76	1.00E-04	1.10E-03	*GLI3*
			HA	A	0.113	0.214	0.08	1.25	5.22E-06	2.33E-05	

			AA	A	0.018	0	0.021	0.87	0.41	0.26	
rs77379983			All	A	0.06	0.129	0.04	1.26	2.05E-08*	3.16E-07	
	7	42221815	EA	A	0.012	0.037	0.006	1.61	4.00E-04	4.40E-03	*GLI3*
			HA	A	0.113	0.214	0.08	1.25	6.31E-06	2.55E-05	
			AA	A	0.014	0	0.015	0.87	0.49	0.37	
rs2058020			All	A	0.06	0.129	0.04	1.26	2.96E-08*	4.20E-07	
	7	42226656	EA	A	0.015	0.044	0.008	1.52	5.20E-04	3.50E-03	*GLI3*
			HA	A	0.113	0.223	0.086	1.24	8.37E-06	3.16E-05	
			AA	A	0.036	0.036	0.036	1	0.99	0.91	
rs76257295			All	A	0.07	0.142	0.048	1.23	4.66E-08*	2.51E-07	
	7	42237521	EA	T	0.014	0.037	0.008	1.59	3.68E-04	8.70E-03	*GLI3*
			HA	T	0.113	0.218	0.085	1.24	8.94E-06	5.70E-05	
			AA	T	0.014	0	0.015	0.87	0.49	0.37	
			All	T	0.06	0.135	0.044	1.24	5.57E-08*	8.62E-07	

top SNPs for HA only

rs188038522											
	7	42209806	EA	C	0.008	na	na	na	na	na	*GLI3*
			HA	C	0.123	0.228	0.088	1.37	2.27E-06	1.20E-05	
			AA	C	0.018	0	0.021	0.87	0.41	0.26	
rs3801212			All	C	0.066	0.135	0.047	1.23	3.80E-07	2.18E-06	
	7	42207032	EA	G	0.011	0.022	0.008	1.38	0.022	0.097	*GLI3*
			HA	G	0.182	0.291	0.145	1.2	4.40E-06	2.01E-05	
			AA	G	0.34	0.036	0.034	1.02	0.64	0.76	
			All	G	0.13	0.193	0.116	1.14	8.30E-06	3.63E-05	

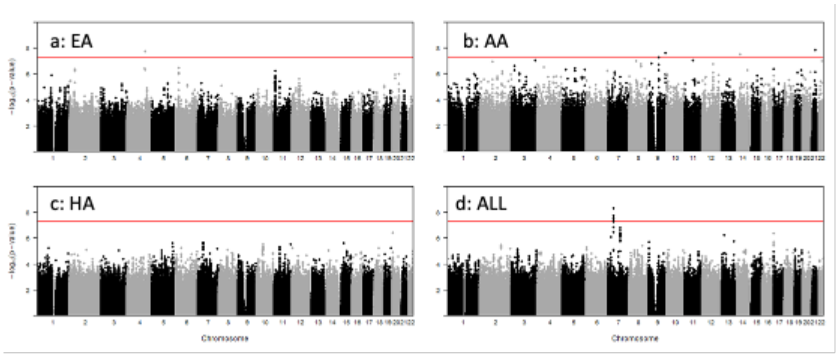

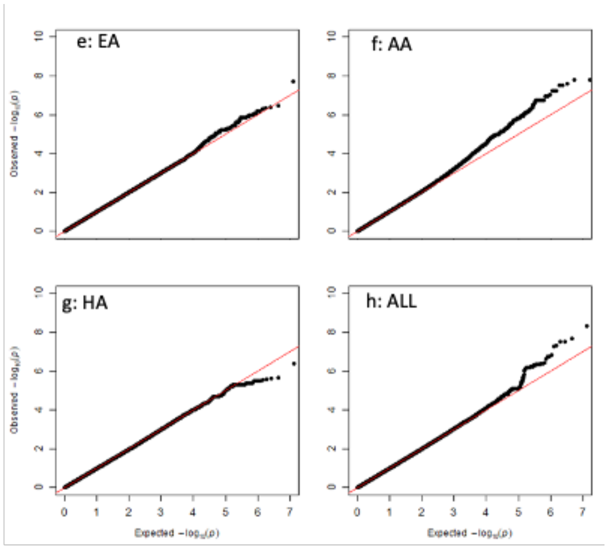

Supplementary Figure 1: *GLI3* variation is associated with ROP severity in a multiethnic cohort.

Manhattan plot (a-d) and QQ plot (e-h) of genome-wide association with ROP Stage stratified by ethnic and racial groups. The red line on Manhattan plot indicates genome-wide significance ($p < 5x10^{-8}$). The quantile-quantile (QQ) plot represents all observed p values ordered from largest to smallest against expected values from a chi-squared distribution. This plot can be used to detect systematic differences from expected due to the population stratification.
(EA: European Ancestry; HA: Hispanic American; AA: African American)
