# Supplementary material for: Genome-wide association identifies novel ROP risk loci in a multiethnic cohort
Source: Commun Biol. 2024 Jan 17;7:107. doi: 10.1038/s42003-023-05743-9 (PMC10794688; doi:10.1038/s42003-023-05743-9)
Supplement: Supplementary file 3 — Description of Additional Supplementary Files [file 42003_2023_5743_MOESM3_ESM.pdf]

Description of Additional Supplementary Files

File name: Supplementary Data 1
Description: Racial and Ethnic population analysis for top ROP-associated SNPs.
